# Genome Sequence of the Fish Pathogen *Yersinia ruckeri* Strain 150, Isolated from Diseased Rainbow Trout

**DOI:** 10.1128/genomeA.01331-16

**Published:** 2016-12-01

**Authors:** Desirée Cascales, José A. Guijarro, Pilar Reimundo, Ana I. García-Torrico, Jessica Méndez

**Affiliations:** Área de Microbiología, Departamento de Biología Funcional, IUBA, Universidad de Oviedo, Spain

## Abstract

We present here the draft genome of a pathogenic *Yersinia ruckeri* strain, isolated from rainbow trout (*Oncorhynchus mykiss*) affected by enteric redmouth disease. The chromosome has 3,826,775 bp, a GC content of 46.88%, and is predicted to contain 3,538 coding sequences. The data will be useful for comparative pathogenicity studies.

## GENOME ANNOUNCEMENT

*Yersinia ruckeri*, a Gram-negative bacterium, is the etiological agent of enteric redmouth disease (ERM), a hemorrhagic septicemia in fish. Since its first isolation from a rainbow trout in Idaho in the 1950s (1), this microorganism has spread to many countries infecting different fish species (2), leading to significant economic losses in salmonid aquaculture.

Species classifications distinguish four O-serotypes with different subgroups: serotypes O1 (subgroup a and b), O2 (subgroups a, b, and c), O3, and O4 (3). Other intraspecific classification subdivides *Y. ruckeri* strains into biotypes 1 and 2 (4). The majority of epizootics in fish farms are produced by serotype O1a, biotype 1 strains.

Since the 1970s commercial vaccines against ERM, consisting of inactivated *Y. ruckeri* cells of serotype O1, biotype 1, have been developed. Initially, vaccination was very useful to control the appearance of the disease, but, recently, ERM outbreaks in vaccinated fish have been reported. These were produced by serotype O1, biotype 2 *Y. ruckeri* strains (5–7). Nowadays, a commercialized bivalent vaccine is available that includes both biotype 1 and 2 strains. Despite the effectiveness of this vaccine, it is essential to investigate the virulence factors of this pathogen (8) since new vaccines or treatments could be necessary in the future.

Presently, apart from the genome sequence of the type strain ATCC 29473, only seven draft *Y. ruckeri* genomes are available. These correspond to heterogeneous strains of O1 and O2 serotypes that were isolated from different hosts like rainbow trout (9), salmon (10), or channel catfish (11).

As a way to increase the knowledge of this fish pathogen, we report here the genome sequence of the serotype O1 *Y. ruckeri* strain 150, isolated in Denmark from an ERM-affected rainbow trout. Genomic DNA was extracted using a GenElute bacterial genomic DNA kit (Sigma Aldrich and Co.). High-throughput Illumina sequencing technology was used to conduct paired-end sequencing of genomic DNA and construct a ~700-bp library with 537 Mb of raw data. The reads were assembled using the SOAPdenovo alignment tool (12) into 49 scaffolds with sizes ranging from 502 bp to 572,189 bp. The draft genome of *Y. ruckeri* strain 150 is 3,826,775-bp long with a GC content of 46.88%. Genome functional annotation performed with RAST (13) found 3,538 predicted coding sequences, four rRNAs, and 21 tRNAs. Using the Tandem Repeats Finder database (14), 87 tandem repeats, repeated from 2 to 8.5 times, were found. CRISPR Finder (15) did not show any clustered regularly interspaced short palindromic repeats. Also, four prophage regions where identified by PHAST (16), of which one region was intact, two regions were incomplete, and one region was questionable.

The comparative genomic analysis of *Y. ruckeri* annotated genomes could be useful to gain more insight into the general virulence mechanisms of this pathogen or to identify those genes responsible for host-specific adaptations. It is also possible that the comparative analysis will expose differences among strains that could justify vaccination failures.

### Accession number(s).

This whole-genome shotgun project has been deposited at DDBJ/ENA/GenBank under the accession number MKFJ00000000. The version described in this paper is the first version, MKFJ01000000.

## References

[1] Ross AJ, Rucker RR, Ewing WH 1966 Description of a bacterium associated with redmouth disease of rainbow trout (*Salmo gairdneri*). Can J Microbiol 12:763–770. doi:10.1139/m66-103. 6007992

[2] Tobback E, Decostere A, Hermans K, Haesebrouck F, Chiers K 2007 *Yersinia ruckeri* infections in salmonid fish. J Fish Dis 30:257–268. doi:10.1111/j.1365-2761.2007.00816.x. 17501736

[3] Romalde JL, Magariños B, Barja JL, Toranzo AE 1993 Antigenic and molecular characterization of *Yersinia ruckeri*: proposal for a new intraspecies classification. Syst Appl Microbiol 16:411–419. doi:10.1016/S0723-2020(11)80274-2.

[4] Davies RL, Frerichs GN 1989 Morphological and biochemical differences among isolates of *Yersinia ruckeri* obtained from wide geographical areas. J Fish Dis 12:357–365. doi:10.1111/j.1365-2761.1989.tb00324.x.

[5] Austin DA, Robertson PA, Austin B 2003 Recovery of a new biogroup of *Yersinia ruckeri* from diseased rainbow trout (*Oncorhynchus mykiss*, Walbaum). Syst Appl Microbiol 26:127–131. doi:10.1078/072320203322337416. 12747420

[6] Arias CR, Olivares-Fuster O, Hayden K, Shoemaker CA, Grizzle JM, Klesius PH 2007 First report of *Yersinia ruckeri* biotype 2 in the USA. J Aquat Anim Health 19:35–40. 18236630 10.1577/H06-011.1

[7] Fouz B, Zarza C, Amaro C 2006 First description of non-motile *Yersinia ruckeri* serovar I strains causing disease in rainbow trout, *Oncorhynchus mykiss* (Walbaum), cultured in Spain. J Fish Dis 29:339–346. doi:10.1111/j.1365-2761.2006.00723.x. 16768714

[8] Fernández L, Méndez J, Guijarro JA 2007 Molecular virulence mechanisms of the fish pathogen *Yersinia ruckeri*. Vet Microbiol 125:1–10. doi:10.1016/j.vetmic.2007.06.013. 17651924

[9] Nelson MC, LaPatra SE, Welch TJ, Graf J 2015 Complete genome sequence of *Yersinia ruckeri* strain CSF007-82, etiologic agent of red mouth disease in salmonid fish. Genome Announc 3(1):e01491-14. doi:10.1128/genomeA.01491-14. PMC4319512 25635018

[10] Navas E, Bohle H, Henríquez P, Grothusen H, Bustamante F, Bustos P, Mancilla M 2014 Draft genome sequence of the fish pathogen *Yersinia ruckeri* strain 37551, serotype O1b, isolated from diseased, vaccinated Atlantic salmon (*Salmo salar*) in Chile. Genome Announc 2(4):e00858-14. doi:10.1128/genomeA.00858-14. 25169862 PMC4148730

[11] Wang KY, Liu T, Wang J, Chen DF, Wu XJ, Jiang J, Liu JX 2015 Complete genome sequence of the fish pathogen *Yersinia ruckeri* strain SC09, isolated from diseased *Ictalurus punctatus* in China. Genome Announc 3(1):e01327-14. doi:10.1128/genomeA.01327-14. PMC4290980 25573927

[12] Luo R, Liu B, Xie Y, Li Z, Huang W, Yuan J, He G, Chen Y, Pan Q, Liu Y, Tang J, Wu G, Zhang H, Shi Y, Liu Y, Yu C, Wang B, Lu Y, Han C, Cheung DW, Yiu SM, Peng S, Xiaoqian Z, Liu G, Liao X, Li Y, Yang H, Wang J, Lam TW, Wang J 2012 SOAPdenovo2: an empirically improved memory-efficient short-read *de novo* assembler. GigaScience 1:18. doi:10.1186/2047-217X-1-18. 23587118 PMC3626529

[13] Brettin T, Davis JJ, Disz T, Edwards RA, Gerdes S, Olsen GJ, Olson R, Overbeek R, Parrello B, Pusch GD, Shukla M, Thomason JA, Stevens R, Vonstein V, Wattam AR, Xia F 2015 RAST*tk*: a modular and extensible implementation of the RAST algorithm for building custom annotation pipelines and annotating batches of genomes. Sci Rep 5:8365. doi:10.1038/srep08365. 25666585 PMC4322359

[14] Benson G 1999 Tandem repeats finder: a program to analyze DNA sequences. Nucleic Acids Res 27:573–580. doi:10.1093/nar/27.2.573. 9862982 PMC148217

[15] Grissa I, Vergnaud G, Pourcel C 2007 CRISPRFinder: a web tool to identify clustered regularly interspaced short palindromic repeats. Nucleic Acids Res 35:W52–W57. doi:10.1093/nar/gkm360. 17537822 PMC1933234

[16] Zhou Y, Liang Y, Lynch KH, Dennis JJ, Wishart DS 2011 PHAST: a fast phage search tool. Nucleic Acids Res 39:W347–W352. doi:10.1093/nar/gkr485. 21672955 PMC3125810

